# Patient Continued Use of Online Health Care Communities: Web Mining of Patient-Doctor Communication

**DOI:** 10.2196/jmir.9127

**Published:** 2018-04-16

**Authors:** Bing Wu

**Affiliations:** ^1^ School of Economics and Management Tongji University Shanghai China

**Keywords:** health information management, health communication, information literacy, social networking

## Abstract

**Background:**

In practice, online health communities have passed the adoption stage and reached the diffusion phase of development. In this phase, patients equipped with knowledge regarding the issues involved in health care are capable of switching between different communities to maximize their online health community activities. Online health communities employ doctors to answer patient questions, and high quality online health communities are more likely to be acknowledged by patients. Therefore, the factors that motivate patients to maintain ongoing relationships with online health communities must be addressed. However, this has received limited scholarly attention.

**Objective:**

The purpose of this study was to identify the factors that drive patients to continue their use of online health communities where doctor-patient communication occurs. This was achieved by integrating the information system success model with online health community features.

**Methods:**

A Web spider was used to download and extract data from one of the most authoritative Chinese online health communities in which communication occurs between doctors and patients. The time span analyzed in this study was from January 2017 to March 2017. A sample of 469 valid anonymous patients with 9667 posts was obtained (the equivalent of 469 respondents in survey research). A combination of Web mining and structural equation modeling was then conducted to test the research hypotheses.

**Results:**

The results show that the research framework for integrating the information system success model and online health community features contributes to our understanding of the factors that drive patients' relationships with online health communities. The primary findings are as follows: (1) perceived usefulness is found to be significantly determined by three exogenous variables (ie, social support, information quality, and service quality; *R*^2^=0.88). These variables explain 87.6% of the variance in perceived usefulness of online health communities; (2) similarly, patient satisfaction was found to be significantly determined by the three variables listed above (*R*^2^=0.69). These variables explain 69.3% of the variance seen in patient satisfaction; (3) continuance use (dependent variable) is significantly influenced by perceived usefulness and patient satisfaction (*R*^2^=0.93). That is, the combined effects of perceived usefulness and patient satisfaction explain 93.4% of the variance seen in continuance use; and (4) unexpectedly, individual literacy had no influence on perceived usefulness and satisfaction of patients using online health communities.

**Conclusions:**

First, this study contributes to the existing literature on the continuance use of online health communities using an empirical approach. Second, an appropriate metric was developed to assess constructs related to the proposed research model. Additionally, a Web spider enabled us to acquire objective data relatively easily and frequently, thereby overcoming a major limitation of survey techniques.

## Introduction

### Background

Research indicates that online health communities (OHCs) have become major sources of health information for the general public and have dramatically changed health information seeking and exchange [1]. Individuals seek online health information for various reasons such as quick and easy access, anonymity or privacy, the variety of available information, cost-effectiveness, and improved communication. Thus, health information acquired online can increase people’s knowledge and further develop personal skills and abilities [2].

In practice, OHCs have passed the adoption stage to reach the diffusion phase of development. In this phase, patients equipped with knowledge regarding health care issues are capable of switching between different communities to maximize their OHC activities. OHCs employ doctors to answer patient questions, and high quality OHCs are more likely to be acknowledged by patients. Therefore, one important question that must be addressed is: what factors drive patients to maintain ongoing relationships with OHCs in which communication occurs between doctors and patients? However, this important question has received limited scholarly attention [3].

### Objective

The goal of this study was to examine the factors that drive patients to perpetuate ongoing relationships with OHCs in which communication occurs between doctors and patients, by integrating the information system success model (ISSM) and OHC features. Methodologically, the study uses a combination of Web mining and structural equation modeling (SEM) [4] to analyze data captured by a Web spider from one of the most authoritative OHCs in China. This approach can circumvent many of the problems associated with survey data, such as expense and the need for an acceptable response rate.

## Methods

### Research Model

An OHC is a group of individuals who interact in health and wellness-related virtual communities to seek information, assistance, emotional support, and communication opportunities [5]. Many benefits of OHC participation have been identified, including increased support, perceived empathy, optimism, reduced levels of stress, depression, and psychological trauma [6]. Compared with other online communities, OHCs aim to provide various types of social support [7]. Thus, individual literacy and social support are two important OHC features [8]. In recent years, OHC research has increasingly begun to emphasize relationship-building [9] and psychological empowerment [10]. Although these studies have revealed various interesting findings, the factors that govern the patient continuance use of OHCs remain unknown [3].

As a critical indicator of information system (IS) success, continuance use is essential for realizing the value of IS within organizations [11]. Therefore, the measurement of IS success is important for assessing the effectiveness of IS governance and IS investments. Previous research has identified IS success measures 12. It has been proposed that a system can be evaluated in terms of quality measures such as information quality, system quality, and service quality. These characteristics affect subsequent use and user satisfaction, and contribute to the belief that certain benefits will be achieved by using the system [12].

The validity of the ISSM in various contexts, which has been demonstrated through a variety of empirical studies, indicates that ISSM are well accepted by IS scholars and useful for practitioners [13]. So, it is assumed that the updated ISSM can be adapted for system success measurement in the OHC context [14]. Considering that system quality has been an essential prerequisite for information systems, especially for OHCs [15], we used measures related to quality (ie, information quality and service quality).

**Figure 1:**
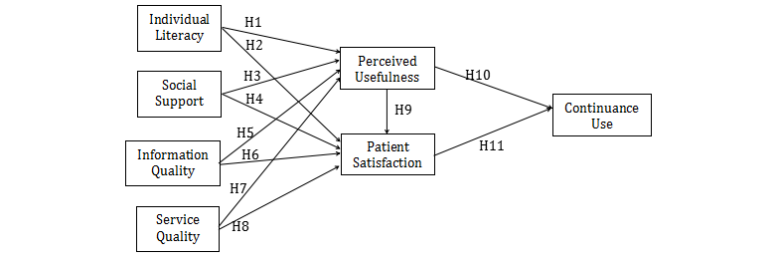
Proposed research model.

In summary, the aim of this study was to develop a research model integrating the ISSM and OHC features to study which factors drive patients to maintain ongoing relationships with OHCs in which communication occurs between doctors and patients. The proposed model identifies several attributes as predictors of a patient’s OHC continuance use. The relationships between the constructs are depicted in the conceptual model demonstrated in Figure 1. The continuance use expresses the likelihood that patients will continue to visit and participate in an OHC. Patient satisfaction and perceived usefulness are primarily subjective concepts influenced by individual literacy, social support, information quality, and service quality. Because no reliable measures developed by Web mining are found in the literature [16], we adjusted and developed a metric to measure constructs in the proposed model.

#### Individual Literacy–Related Hypotheses

Although the current use of OHCs is extensive and their possible benefit for the public is substantial, website limitations prevent patients from exploiting the full potential of OHCs. Problems associated with retrieving information from OHCs tend to involve language, literacy, education, technology, and cultural barriers [17].

The relationship between individual literacy and perceived usefulness is of interest to many scholars [18]. Three types of literacy (ie, information literacy, media literacy, and health literacy) are related to understanding and acting on individual literacy in this study. They can be measured by Web mining patients’ posts [19]. We define patient post as a new thread posted by a patient that can be followed by replies from doctors and additional questions from the patient.

##### Information Literacy

Information literacy is defined as a set of skills that are necessary to identify, access, evaluate, and use information effectively [20]. It is important for individuals who wish to access health information and relates to their ability to share content [21]. The text length of a patient’s post [22] is an important indicator of the patient’s information literacy.

**Figure 2:**
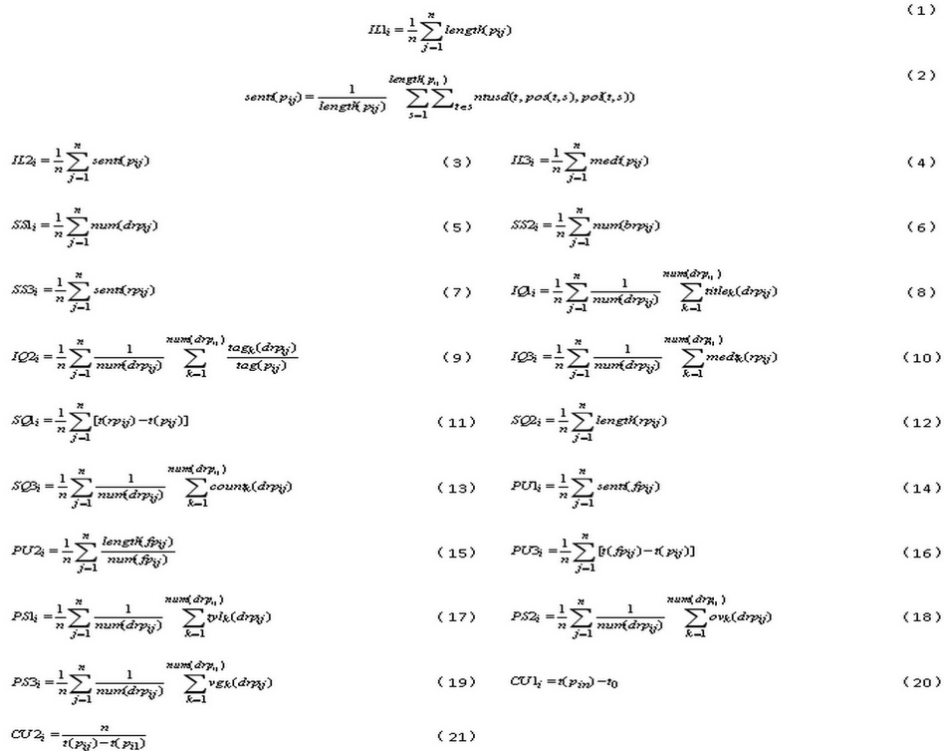
Equations.

Thus, the information literacy of a patient *i* (ie, *IL*1_i_) could be evaluated by the average text length of each post (equation 1, Figure 2), where *length*(*p*_ij_) is the text length of the *j*^th^ post *p*_ij_ by patient *i* and *n* is the total number of posts by patient *i* during a certain time interval.

##### Media Literacy

Media literacy is defined as the ability to evaluate and judge the accuracy of media information [23]. The sentiment of a patient’s post [24, which refers to feelings and emotions expressed by a patient through their post, can be used as a critical indicator of the patient’s media literacy. Sentiment analysis can be used to systematically quantify subjective information from a patient’s post. We use the Chinese sentiment polarity dictionary, National Taiwan University Semantic Dictionary (NTUSD), developed by Taiwan University, to identify the sentiment of each post *p*_ij_ of patient *i* as a negative, neutral, or positive emotion (equation 2, Figure 2) [25], where the sentiment score *senti*(*p*_ij_) is the *j*^th^ post *p*_ij_ by patient *i*, *length*(*p*_ij_) is the text length of the *j*^th^ post *p*_ij_ by patient *i,s* is number of sentences in the *j*^th^ post *p*_ij_ by patient *i*, and *t* is sentiment expression terms in sentences, *pos*(*t,s*) is part of *t* in sentence *s*, *pol*(*t,s*) polarity of *t* in sentence *s*, and *ntusd(t, pos(t,s), pol(t,s))* is the NTUSD intensity value for term *t* based on its sentence part *pos*(*t,s*) and polarity *pol*(*t,s*). The media literacy of patient *i* (ie, *IL*2_i_) could be calculated as the average sentiment of each post (equation 3, Figure 2), assuming the total number of posts by patient *i* is *n* during a certain time interval.

##### Health Literacy

Health literacy has been defined as the ability to read, comprehend, and act upon health information [26]. The number of medical terms in a patient’s post can be an indicator of the patient’s proficiency in health literacy [27]. We began by extracting the medical terminology from the tag in the OHC. Next, we counted the examples of these terminologies contained in each post of patient *i*,. Health literacy of patient *i* (ie, *IL3*_i_) could then be calculated as the average number of medical terms in each post (equation 4; Figure 2), where *medt*(*p*_ij_) is the number of medical terms in the *j*^th^ post *p*_ij_ by patient *i*, and *n* is the total number of posts by patient *i* during a certain time interval.

##### Individual Literacy

Generally, individual literacy is directly related to the ability to communicate with others [28] and to express individual needs. This, in turn, affects the patient’s perceived usefulness of and satisfaction with OHCs. Accordingly, we propose the following research hypotheses:

Hypothesis 1: individual literacy has a positive effect on perceived usefulness of OHCs.

Hypothesis 2: individual literacy has a positive effect on patient satisfaction when using OHCs.

#### Social Support–Related Hypotheses

The social support aspect is used to assess whether patients can benefit from social relationships in the OHC context [29]. Social support in OHCs refers to the verbal and nonverbal communication exchanged between recipients and providers that reduces uncertainty regarding the situation or the relationship, and functions to enhance a perception of personal control in one’s experience [30].

Three types of social support (ie, companionship support, informational support, and emotional support) [31] are examined in this study. They can be measured by Web mining of doctors’ replies to patients’ posts.

##### Companionship Support

Companionship support gives someone a sense of social belonging, through the presence of companions to engage in shared social activities [32]. Companionship support in OHCs refers to communications of opinions or facts relevant to current health-related difficulties (eg, advice, personal feedback, and information) that may provide a solution to a problem. Such support also increases information sharing, which in turn leads to a better patient-doctor experience. Thus, companionship support to patient *i* (ie, *SS*1_i_) could be calculated as the average number of doctors who replied to each post by the patient (equation 5, Figure 2), where *num*(*drp*_ij_) is the total number of doctors who replied to the *j*^th^ post *p*_ij_ by patient *i*, and *n* is the total number of posts by patient *i* during a certain time interval.

##### Informational Support

Informational support refers to advice, guidance, suggestions, or useful information provided to someone. Informational support in OHCs involves a doctor-patient interaction where the former assists latter with managing particular questions and uncertainties regarding a given situation in a highly supportive manner [33]. The number of best answers provided by doctors is an important indicator of valuable support to patients. Valuable support to patient *i* (ie, *SS2*_i_) could be calculated as the average number of best answers provided by doctors to each post by the patient (equation 6, Figure 2), where *num*(*brp*_ij_) is the total number of best answers provided by doctors to the *j*^th^ post *p*_ij_ by patient *i*, and *n* is the total number of posts by patient *i* during a certain time interval.

##### Emotional Support

Patients in an OHC are influenced by the emotional support they receive and the quality of information and companionship they are provided [34]. Interaction through OHCs can increase emotional support and lead to a sense of self-esteem in patients [35]. Studies have reprted on the positive impact of such support on overall health outcomes, health education, and patient empowerment [36]. Emotional support to patient *i* (ie, *SS3*_i_) could be calculated as the average reply sentiment of the doctors to each post (equation 7, Figure 2), where sentiment is calculated according to equation 2 in Figure 2, *senti*(*rp*_ij_) is the reply sentiment of the doctors to the *j*^th^ post *p*_ij_ by patient *i*, and *n* is the total number of posts by patient *i* during a certain time interval.

##### Social Support

The social support of OHCs is credited with many positive health outcomes such as increased immune system strength and reduced stress and depression [37]. Here, we suggest that positive social support will translate into positive perceptions of OHCs. Thus, the following hypotheses are proposed:

Hypothesis 3: social support has a positive effect on perceived usefulness of OHCs.

Hypothesis 4: social support has a positive effect on patient satisfaction when using OHCs.

#### Information Quality–Related Hypotheses

Information quality in OHCs refers to content issues and includes the completeness, accuracy, format, and currency aspects of the information [38]. Three types of information quality (ie, information reliability, information relevancy, and information consistency) [39] are identified in this study that can be measured by Web mining doctors’ replies to patients’ posts.

##### Information Reliability

Information reliability is, literally, the extent to which an individual can rely on the source of the information [40]. The official title of doctors certified by a national agency with uniform standards is a guarantee of reliable information sources. Thus, the reliability of information sources for patient *i* (ie, *IQ*1_i_) could be calculated as the average title of the doctors who replied to each post (equation 8, Figure 2), where *title*(*drp*_ij_) is the academic title of the *k*^th^ doctors who replied to the *j*^th^ post *p*_ij_ by patient *i*, *num*(*drp*_ij_) is the total number of doctors who replied to the *j*^th^ post *p*_ij_ by patient *i,* and *n* is the total number of posts by patient *i* during a certain time interval. A scale of 1 to 5 was used to normalize the job titles (1=assistant doctor, 2=resident doctor, 3=chief doctor, 4=associate archiater, and 5=archiater).

##### Information Relevancy

Information relevancy in OHCs means that information is applicable and helpful for health-related goals of patients [41]. Thus, we calculated the degree of relevancy between the tags of each post field of patient *i* replied to by doctors and the expertise tag of each replying doctor. Then, the information relevancy to patient *i* (ie, *IQ*2_i_) could be calculated as the average number of the relevancy degree (equation 9, Figure 2), where *tag*_k_(*drp*_ij_) divided by *tag*(*p*_ij_) is the number of the relevancy degree of the *k*^th^ doctors who replied to the *j*^th^ post *p*_ij_ by patient *i*, *num*(*drp*_ij_) is the total number of doctors who replied to the *j*^th^ post *p*_ij_ by patient *i*, and *n* is the total number of posts by patient *i* during a certain time interval.

##### Information Consistency

Information consistency in OHCs denotes that information is always presented in the same format and is in line with previous information [42]. The number of examples of medical terminology in a doctor’s reply could be a key indicator of information consistency in OHCs. On the basis of the medical terminology extracted from the tag in the OHC, we counted the number of medical terms in each reply to a post. Therefore, the information consistency to patient *i* (ie, *IQ*3_i_)could be calculated as the average number of medical terms in the replies of doctors to each post (equation 10, Figure 2), where *med*(*rp*_ij_) is the number of medical terms in the replies of *k*^th^ doctors who answered the *j*^th^ post *p*_ij_ by patient *i*, *num*(*drp*_ij_) is the total number of doctors who replied to the *j*^th^ post *p*_ij_ by patient *i*, and *n* is the total number of posts by patient *i* during a certain time interval.

##### Information Quality

As a necessary measure of success, OHC information quality is a highly vital factor that affects the patient’s online behavior. This factor indicates the importance of quality online information and references [43]; and information quality significantly affects perceived usefulness [12]. Thus, we propose the following research hypotheses:

Hypothesis 5: information quality has a positive effect on perceived usefulness of OHCs.

Hypothesis 6: information quality has a positive effect on patient satisfaction when using OHCs.

#### Service Quality–Related Hypotheses

OHC service quality is defined by the feelings of patients regarding the level of service while browsing and interacting within the community [44]. Three types of service quality (ie, service responsiveness, service effort, and service empathy) are identified in this study that can be measured by Web mining doctors’ replies to patients’ posts.

##### Service Responsiveness

Service responsiveness in OHCs refers to the willingness of doctors to help patients with prompt service. Thus, service responsiveness [45] of doctors to patient *i* (ie, *SQ*1_i_) could be measured as the average time taken for the first reply to be posted to the *j*^th^ post [*t*(*rp*_ij_)−*t*(*p*_ij_)] as shown in Figure 2 (equation 11), assuming the total number of posts by patient *i* is *n* during a certain time interval.

##### Service Effort

Service effort is defined in OHCs as the amount of time and energy the doctor invests in replying to patients [46]. Thus, service effort exerted in responsiveness by doctors to patient *i* (ie, *SQ*2_i_) could be evaluated by the average text length of replies to each post [22], as shown in Figure 2 (equation 12), where *length*(*rp*_ij_) is the text length of replies to the *j*^th^ post *p*_ij_ by patient *i*, and *n* is the total number of posts by patient *i* during a certain time interval.

##### Service Empathy

Service empathy in the OHC means that the doctors provide individualized attention to the patients [47]. The average number of patients to whom doctors have replied could be used as a critical indicator of service empathy. Thus, the service empathy [48] of doctors to patient *i* (ie, *SQ*3_i_) could be evaluated by the average number of patients to whom doctors have replied (equation 13, Figure 2), where *count*_k_(*drp*_ij_) is the total number of patients to whom the *k*^th^ doctor replied, in which the *k*^th^ doctor is the one who replied to *j*^th^ post *p*_ij_ by patient *i*, *num*(*drp*_ij_) is the total number of doctors who replied to the *j*^th^ post *p*_ij_ by patient *i*, and *n* is the total number of posts by patient *i* during a certain time interval *i*.

##### Service Quality

Service quality at OHCs differs from the traditionally studied service quality in IS research, which focuses on the services of organizations [49]. OHCs not only support the creation, exchange, and perception of information but also create a virtual community to support the use of collaborative and interactive services. In recent years, there have been a number of studies on how service quality affects perceived usefulness and patient satisfaction in OHCs [50]. Several studies have observed that service quality has a significant effect on patient satisfaction [50].

This study proposes that service quality influences the perceived usefulness and patient satisfaction of OHCs, which, in turn, influences continuance use. Thus, the following hypotheses are proposed:

Hypothesis 7: service quality has a positive effect on perceived usefulness of OHCs.

Hypothesis 8: service quality has a positive effect on patient satisfaction when using OHCs.

#### Perceived Usefulness–Related Hypotheses

On the basis of its definition as capable of being used advantageously, perceived usefulness is explained as the degree to which a person believes that using a particular system would enhance his or her job performance [51]. The perceived usefulness of an OHC can be described as the extent to which a patient believes that an OHC can be useful in achieving health-related goals.

A patient post can be followed by replies from doctors, and then be followed by additional questions from the patient. The feedback provided through this back-and-forth dialogue adds to the perceived usefulness of patients [17,52]. Thus, three types of perceived usefulness (ie, feedback emotion, feedback effort, and feedback time) are identified in this study that can be measured by Web mining patients’ additional questions.

##### Feedback Emotion

As indicators of whether or not motives are satisfied in OHCs, emotions in additional questions show how patients feel about replies from doctors. Thus, emotions in patient feedback *i* (ie, *PU*1_i_) could be calculated as the average sentiment of additional questions to each post (equation 14, Figure 2), where *senti*(*fp*_ij_) is the sentiment of each additional question of the *j*^th^ post *p*_ij_ by patient *i*, and *n* is the total number of posts by patient *i* during a certain time interval.

##### Feedback Effort

The patient feedback effort in OHCs is the amount of time and energy the patient invests in feedback to doctors. The patients can signal their perceived usefulness by feedback efforts. Thus, the feedback effort of patient *i* (ie, *PU*2_i_) could be calculated as the average ratio of length to number of additional questions to each post (equation 15, Figure 2), where *num*(*fp*_ij_) is the total number of additional questions of the *j*^th^ post *p*_ij_ by patient *i*, *length*(*fp*_ij_) the text length of additional questions of the *j*^th^ post *p*_ij_ by patient *i* and, *n* is the total number of posts by patient *i* during a certain time interval.

##### Feedback Time

Feedback time in OHCs is the total amount of time it takes for patients to respond to replies from doctors. Thus, the feedback time of patient *i* (ie, *PU*3_i_) could be evaluated by the average time taken before posting the first additional question following a post (equation 16, Figure 2), where *t*(*fp*_ij_)−*t*(*p*_ij_) is the time interval until the first additional question to the *j*^th^ post *p*_ij_ by patient *i*, and *n* is the total number of posts by patient *i* during a certain time interval.

##### Perceived Usefulness

A system high in perceived usefulness is one that a user believes offers a positive use-performance relationship [53]. Several prior studies suggest that perceived usefulness is the antecedent of overall customer satisfaction. Additionally, perceived usefulness is a construct that has been repeatedly shown to influence attitude and is a direct determinant of continued IS use [54]. Thus, the following hypotheses are proposed:

Hypothesis 9: perceived usefulness has a positive effect on patient satisfaction when using OHCs.

Hypothesis 10: perceived usefulness has a positive effect on the continuance use of OHCs.

#### Patient Satisfaction–Related Hypotheses

In the OHC context, patient satisfaction describes the feelings, attitudes, and expectations of patients who perceive that they have received good services from an OHC and are likely to revisit. Since the 1980s, user satisfaction has received considerable attention in the IS literature and remains a focus of considerable research interest [55]. The quality of a user’s experience and satisfaction with a website has been used in recent research as determinants of success [56].

Patients can express opinions to doctors conveniently and easily in OHCs; thus, their satisfactions can be broadly recognized by the total number of public thank-you letters, online votes, and virtual gifts to doctors. Therefore, three types of patient satisfaction are identified in this study (ie, satisfaction via thank-you letters, satisfaction via online votes, and satisfaction via virtual gifts).

##### Satisfaction via Thank-You Letters

Thank-you letters are written by patients to doctors to express appreciation in OHCs. Thus, satisfaction via thank-you letters of patient *i* (ie, *PS*1_i_) could be evaluated by the average number of thank-you letters received by doctors who replied to a post (equation 17, Figure 2), where *tyl*_k_(*drp*_ij_) is the total number of thank-you letters for the *k*^th^ doctor who replied to the *j*^th^ post *p*_ij_ by patient *i*, *num*(*drp*_ij_) is the total number of doctors who replied to *j*^th^ post *p*_ij_ by patient *i*, and *n* is the total number of posts by patient *i* during a certain time interval.

##### Satisfaction via Online Votes

Online votes show praise given by patients to doctors in OHCs. Thus, satisfaction via online votes of patient *i* (ie, *PS*2_i_) could be evaluated by the average number of online votes to doctors who replied to posts (equation 18, Figure 2), where *ov*_k_(*drp*_ij_) is the total number of online votes for the *k*^th^ doctor who replied to the *j*^th^ post *p*_ij_ by patient *i*, *num*(*drp*_ij_) is the total number of doctors who replied to *the j*^th^ post *p*_ij_ by patient *i*, and *n* is the total number of posts by patient *i* during a certain time interval.

##### Satisfaction via Virtual Gifts

Virtual gifts are sent to doctors by patients in OHCs. Thus, satisfaction via virtual gifts of patient *i* (ie, *PS*3_i_) could be evaluated by the average number of virtual gifts given to doctors who replied to posts (equation 19, Figure 2), where *vg*_k_(*drp*_ij_) is the total number of virtual gifts for the *k*^th^ doctor who replied to the *j*^th^ post *p*_ij_ by patient *i*, *num*(*drp*_ij_) is the total number of doctors who replied to the *j*^th^ post *p*_ij_ by patient *i*, and *n* is the total number of posts by patient *i* during a certain time interval.

##### Patient Satisfaction

As DeLone and McLean [12] suggest, a positive use experience will result in increased user satisfaction. Similarly, increased patient satisfaction will result in increased continuance use [57]. Because user satisfaction is a significant influencer of user retention, it is often regarded as the most useful and easiest way of evaluating an IS [14]. Thus, the following hypothesis is proposed:

Hypothesis 11: patient satisfaction has a positive effect on the continuance use of OHCs.

#### Continuance Use

Typically, continuance use illustrates patients’ behavior to continue using OHCs [58,59]. Thus, two types of patient continuance use are identified in this study (ie, membership length and frequency of posts from patients).

The membership length of patient *i* (ie, *CU*1_i_) could be calculated as the time span from registration to the present (equation 20, Figure 2).

The posting frequency of patient *i* (ie, *CU*2_i_) could be calculated as *n* posts during *t*(*p*_ij_)−*t*(*p*_i1_) as shown in Figure 2 (equation 21), assuming the total number of posts by patient *i* is *n* during a certain time interval.

### Data Collection

The objective of this study was to understand the factors that drive patients to maintain ongoing relationship with OHCs in which communication occurs between doctors and patients. Although survey instruments are often used in such circumstances, data are difficult to obtain, and data availability and sample size problems may occur. Therefore, this study collected objective data directly from OHCs by using a Web spider.

To test our hypotheses, we captured data from a Chinese OHC (the club.xywy website). This OHC is one of the most authoritative forums in China that connects individuals with health problems with doctors who can help them solve these problems.

In this study, Locoy Spider (Hefei loy Information Technology Co, Ltd China) was employed to download and extract data. This software is freely available and can be downloaded from the Locoy website. The time span analyzed in our study was from January 2017 to March 2017. We preprocessed the data according to the following rules: (1) delete records with no doctor’s reply; (2) delete records not in accordance with the required format; and (3) delete records with advertisements. Finally, 469 valid anonymous patients with 9667 posts were obtained, which is equal to 469 respondents in survey research. Table 1 summarizes the demographics of the patients in the dataset.

### Measure Items in the Dataset

Table 2 summarizes the measure items in the dataset according to research hypotheses. We used z-score to normalize each measure item before data analysis.

### Data Analysis

First, we examined the fitness and construct validity of the proposed measurement model by assessing reliability, convergent validity, and discriminant validity. Then, we examined the structural model to investigate the strength and direction of the relationships among the theoretical constructs.

### Construct Validity

#### Evaluation of Reliability and Convergent Validity

The reliability of all multi-item constructs should exhibit a Cronbach alpha larger than .70. Convergent validity should meet the following criterion: the indicator’s estimated coefficient should be significant with respect to its posited underlying construct factor.

The measurement scales were evaluated using three criteria: all item-factor loadings (k) should be significant and exceed 0.7, the composite reliability (CR) for each construct should exceed 0.7, and the average variance extracted (AVE) for each construct should be larger than 0.5 [60]. Table 3 shows that the item loading, AVE, CR, and Cronbach alpha values for all constructs in the measurement model exceeded the recommended threshold values. In sum, the adequacy of the measurement model indicated that all items were reliable indicators of the hypothesized constructs.

**Table 1:** Demographics characteristics of the patients.

Items		Frequency (n=469), n (%)
**Gender**			
	Male	250 (53.3)
	Female	219 (46.7)
**Age (years)**			
	Under 30	114 (24.3)
	30 or above	355 (75.7)

**Table 2:** Measure items in the dataset.

Construct code and measures			Mean (SD)
**Individual literacy (IL) [20,23,26]**			
	IL1	Patient post text length (to measure information literacy from low to high)	18.8 (15.3)
	IL2	Patient post sentiment (to measure media literacy from low to high)	8.2 (2.4)
	IL3	Number of medical terms in a patient post (to measure health literacy from low to high)	7.7 (3.0)
**Social support (SS) [16,32,34]**			
	SS1	Number of doctors who reply to a patient post (to measure social contact from low to high)	852.7 (309.4)
	SS2	Number of best answers to a patient post (to measure valuable assistance from low to high)	15.1 (9.9)
	SS3	Sentiment of the doctor’s reply to a patient post (to measure emotional support from low to high)	10.7 (3.8)
**Information quality (IQ) [40,41,42,59]**			
	IQ1	Job titles of doctors who reply to a patient post (to measure information reliability from low to high)	3.5 (1.8)
	IQ2	Relevance degree between the expertise of the doctors who reply to a patient post and the field addressed by a patient post (to measure information relevancy from low to high)	5.5 (3.6)
	IQ3	Number of medical terms in the doctor’s reply (to measure information consistency from low to high)	10.2 (8.1)
**Service quality (SQ) [45,46,47]**				
	SQ1	Responsiveness of the doctors to a patient post (to measure service responsiveness from low to high)	4.8 (2.3)
	SQ2	Text length of doctors' replies (to measure service effort from low to high)	20.9 (17.3)
	SQ3	Number of patients replied to by doctors (to measure service empathy from low to high)	189.6 (148.3)
**Perceived usefulness (PU) [17,52,54]**			
	PU1	Sentiment of the patient’s additional questions (to measure feedback emotion from low to high)	8.7 (4.2)
	PU2	Ratio of length to number of the patient’s additional questions (to measure feedback effort from low to high)	13.8 (8.1)
	PU3	Time interval until the patient’s first additional question (to measure feedback time from low to high)	15.1 (13.3)
**Patient satisfaction (PS) [55,56]**			
	PS1	Number of thank-you letters to doctors (to measure satisfaction via thank-you letters from low to high)	3.5 (1.2)
	PS2	Number of online votes awarded to doctors (to measure satisfaction via online votes from low to high)	12.1 (22.3)
	PS3	Number of virtual gifts sent to doctors (to measure satisfaction via virtual gifts from low to high)	16.2 (33.5)
**Continuance use (CU) [58]**			
	CU1	The time span from registration to the present (to measure membership length from low to high)	20.6 (17.1)
	CU2	Frequency of the patient’s posts (to measure posting frequency from low to high)	10.1 (8.1)

#### Discriminant Validity

To test discriminant validity, the average variance shared between a construct and its measures should be larger than the variance shared by the construct and all other constructs in the model [61]. The correlation analysis is shown in Table 4.

#### Goodness of Fit

The structural model was tested to assess how well the model represented the data. We evaluated the following indices [62]: the chi-square test statistic, the goodness-of-fit index (GFI), the normed fit index, the comparative fit index, the Tucker-Lewis index, and the root mean square residual. Table 5 presents the results and recommended values. We conclude that the GFIs displayed the recommended levels, which suggests that the model provided a good fit with the data.

#### Structural Model for Hypothesis Testing

The purpose of the analysis was to determine which factors and how these factors affect the continuance use. SEM using partial least squares (PLS) was applied to test the hypothesized model. Use of SEM is commonly justified in the social sciences because of its ability to impute relationships between unobserved constructs (latent variables) from observable variables, and PLS is a latent SEM technique that uses a component-based approach to estimation [63]. These techniques facilitate the analysis of a structural model that assesses relationships among theoretical constructs and a measurement model that assesses the reliability and validity of measures. The test of the structural model includes estimates of the path coefficients, which indicate the strengths of the relationships between the dependent and independent variables, and values, which represent the amount of variance explained by the independent variables. Together, the path coefficients (ie, the loadings and significance and *R*^2^) indicate how well the data support the hypothesized model.

**Table 3:** Construct reliability and convergent validity.

Construct code		Item loadings	Average variance extracted	Composite reliability	Cronbach alpha
**Individual literacy (IL)**			0.77	0.94	.89
	IL1	0.95			
	IL2	0.89			
	IL3	0.89			
**Social support (SS)**			0.74	0.96	.91
	SS1	0.95			
	SS2	0.91			
	SS3	0.86			
**Information quality (IQ)**			0.76	0.92	.89
	IQ1	0.95			
	IQ2	0.89			
	IQ3	0.87			
**Service quality (SQ)**			0.74	0.96	.85
	SQ1	0.93			
	SQ2	0.85			
	SQ3	0.85			
**Perceived usefulness (PU)**			0.73	0.92	.90
	PU1	0.95			
	PU2	0.89			
	PU3	0.89			
**Patient satisfaction (PS)**			0.82	0.87	.88
	PS1	0.93			
	PS2	0.87			
	PS3	0.86			
**Continuance use (CU)**			0.77	0.93	.86
	CU1	0.91			
	CU2	0.92			

**Table 4:** Interconstruct correlations and discriminant validity.

Constructs	Individual literacy (IL)	Social support (SS)	Information quality (IQ)	Service quality (SQ)	Perceived usefulness (PU)	Patient satisfaction (PS)	Continuance use (CU)
IL	0.85^a^						
SS	0.46	0.83^a^					
IQ	0.26	0.34	0.68^a^				
SQ	0.05	0.22	0.18	0.77^a^			
PU	0.18	0.38	0.24	0.37	0.61^a^		
PS	0.36	0.12	0.10	0.27	0.53	0.74^a^	
CU	0.27	0.19	0.16	0.18	0.15	0.19	0.63^a^

^a^The average variance extracted for the reflective variables is consistently larger than the off-diagonal squared correlations, which suggests satisfactory discriminant validity among variables.

**Table 5:** Overall model t indices for the research model.

Model t indices	Results value	Recommended value
Chi-square or degrees of freedom	1.88	≤3
Goodness-of-fit index	0.93	≥0.9
Normed fit index	0.96	≥0.9
Comparative fit index	0.92	≥0.9
Tucker-Lewis index	0.92	≥0.9
Root mean square residual	0.07	≤0.08

## Results

### Path Analysis

**Figure 3:**
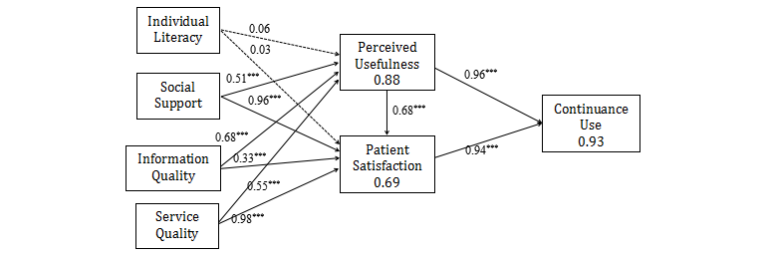
Path analysis. The asterisks *** next to path coefficient values signify *P*<.001.

Figure 3 illustrates the resulting path coefficients of the proposed research model: (1) perceived usefulness is found to be significantly determined by social support, information quality, and service quality (*R*^2^=0.88). These variables explain 87.6% of the variance seen in perceived usefulness; (2) similarly, patient satisfaction is found to be significantly determined by social support, information quality, and service quality (*R*^2^=0.69). These variables explain 69.3% of the variance seen in patient satisfaction; and (3) the dependent variable (continuance use) is significantly determined by perceived usefulness and patient satisfaction (*R*^2^=0.93). That is, the combined effects of perceived usefulness and patient satisfaction explain 93.4% of the variance in continuance use.

A summary of the hypothesis test results of the standardized path coefficients and path significances is provided in Table 6. Most of the paths are significant in the expected direction. Overall, 9 out of 11 hypotheses are supported by the data. The insignificant variables are individual literacy and perceived.

### Influence of Exogenous Variables on Perceived Usefulness and Patient Satisfaction

Unexpectedly, two hypotheses (hypothesis 1 and hypothesis 2), that were supported in previous empirical research, were not supported by our data. The results indicate that individual literacy has no influence on perceived usefulness and patient satisfaction. There are two plausible reasons for this result. First, this may be because nearly 75.7% (355/469) of the sample are older than 30 years, and they seek health information on behalf of others, such as their parents or children. Second, although individual literacy provides the ability to use an OHC, perceived usefulness and patient satisfaction depend on the functionality and characteristics of OHCs. From this perspective, the results are logical.

Social support, information quality, and service quality exert significant direct effects on perceived usefulness and patient satisfaction. Thus, hypothesis 3 to 8 were supported. Generally, social support is an important component of OHCs. In terms of the main effects of social support on perceived usefulness and patient satisfaction, our findings agree with previous research results [64]. Because patients who visit OHCs may regard such communities as repositories of information, information quality and service quality significantly influence perceived usefulness and patient satisfaction. That is, if patients perceive a high quality of information and service, what matters the most when determining their continuance use is whether they have perceived usefulness and satisfaction.

### Influence of Perceived Usefulness and Patient Satisfaction on Continuance Use

Complementing the findings of prior studies [65], perceived usefulness and patient satisfaction had significant effects on continuance use. In addition, the effects of exogenous variables on patient satisfaction are mediated by perceived usefulness.

Overall, these results suggest that increasing social support, informational quality, and service quality may improve the perceived usefulness and patient satisfaction of OHCs, which in turn fosters continuance use within OHCs.

**Table 6:** Model path analysis. OHC: online health community.

Hypotheses (H)	Path coefficient	Support
H1: individual literacy has a positive effect on perceived usefulness	0.06	No
H2: individual literacy has a positive effect on patient satisfaction when using OHCs	0.03	No
H3: social support has a positive effect on perceived usefulness	0.5^a^	Yes
H4: social support has a positive effect on patient satisfaction when using OHCs	0.96^a^	Yes
H5: information quality has a positive effect on perceived usefulness	0.68^b^	Yes
H6: information quality has a positive effect on patient satisfaction when using OHCs	0.33^a^	Yes
H7: service quality has a positive effect on perceived usefulness	0.55^a^	Yes
H8: service quality has a positive effect on patient satisfaction when using OHCs	0.98^a^	Yes
H9: perceived usefulness has a positive effect on patient satisfaction when using OHCs	0.68^a^	Yes
H10: perceived usefulness has a positive effect on the continuance use of OHC	0.96^a^	Yes
H11: patient satisfaction has a positive effect on the continuance use of OHC	0.93^a^	Yes

^a^*P*<.001.

^b^*P*<.01.

## Discussion

### Principal Findings

The results show that the research framework for integrating the ISSM and OHC features contributes to our understanding of the roles certain constructs play in motivating patients to revisit OHCs. Social support, information quality, and service quality exert significant direct effects on perceived usefulness and patient satisfaction. Regarding the influences of perceived usefulness and patient satisfaction, both have significant effects on continuance use of OHC patients. In addition, the effects of social support, information quality, and service quality on patient satisfaction are mediated by perceived usefulness. Overall, these results suggest that increasing social support, informational quality, and service quality may improve the perceived usefulness and patient satisfaction of OHCs, which could in turn positively influence the continuance use of OHC patients. Unexpectedly, individual literacy has no influence on perceived usefulness and satisfaction of OHC patients.

### Limitations and Future Research

Although the results of this study are promising, several limitations must be considered. First, the data analyzed in the study were collected from an OHC in China, in which communications are between patients and doctors. It is unclear whether the results of this study can be generalized to all OHCs, due to the limited number of Chinese OHCs. Therefore, future research should include additional OHCs. Second, this study used cross-sectional data to examine the critical factors that influence patient continuance use of OHCs, which may not fully capture OHC evolution. Future research may conduct longitudinal designs to better understand OHC success.

Despite these limitations, this study has several important implications for future OHC research and interventions. First, Web-tracking techniques can provide more detailed and accurate information on patient behavior patterns, and in turn contribute to improving our understanding of OHC communication mechanisms [66]. The model presented here provides a foundation for future researchers to build on and refine. Second, although we designed a novel metric for the constructs in our model using Web mining, future studies should try to develop more tailored metrics for OHCs. For example, the structural properties of OHCc (eg, size and membership structure) can also be important factors in hypothesis testing.

### Comparison With Prior Work

First, our insights support a reconsideration of the impact of individual literacy on perceived usefulness and patient satisfaction. Because individual literacy does not contribute to perceived usefulness and patient satisfaction, practitioners should invest substantial effort in promoting continuous activity, as patients may seek health information on behalf of others. Because social support [37] contributes to the development of perceived usefulness and patient satisfaction, OHC practitioners should encourage doctors via suitable policies. Similarly, since OHC information quality and service quality strongly influence the development of perceived usefulness and patient satisfaction, it is important to accelerate the accumulation of information resources in terms of defining and archiving existing knowledge [67].

Second, our findings have implications for practitioners concerned with constructing sustainable OHCs that are active and supportive. By showing that perceived usefulness mediates the influence of social support, information quality, and service quality on patient satisfaction and, in turn, on continuance use of OHCs, we emphasize the importance of useful OHC information resources. Therefore, entities responsible for information-resource allocation should use the powerful role that online collectives can play [68].

### Conclusions

The goal of this study was to examine the factors that drive patients to maintain ongoing relationships with OHCs in which communication occurs between doctors and patients. First, this study contributes to the literature by identifying which factors and how these factors influence patient continuance use. Moreover, the measures for the constructs in the proposed model have intuitive explanations and display good performance.

Second, we developed an appropriate metric for constructs in the proposed model. In addition, a Web spider to collect data enabled us to acquire objective data frequently and with relative ease, thereby overcoming a major limitation of survey-based techniques. For example, if a questionnaire alone had been used, we could not control for all variables or claim causal effects regarding sentiment change [69]. Thus, the data collection technique and the SEM method provide new opportunities for measuring people's health behavior in the OHC context [70].

## Abbreviations

AVE: average variance extracted
CR: composite reliability
GFI: goodness-of-fit index
IS: information system
ISSM: information system success model
NTUSD: National Taiwan University Semantic Dictionary
OHC: online health community
PLS: partial least squares
SEM: structural equation modeling
